# Multiple Myeloma and Its Precursor Disease Among Firefighters Exposed to the World Trade Center Disaster

**DOI:** 10.1001/jamaoncol.2018.0509

**Published:** 2018-04-26

**Authors:** Ola Landgren, Rachel Zeig-Owens, Orsolya Giricz, David Goldfarb, Kaznouri Murata, Katie Thoren, Lakshmi Ramanathan, Malin Hultcrantz, Ahmet Dogan, George Nwankwo, Ulrich Steidl, Kith Pradhan, Charles B. Hall, Hillel W. Cohen, Nadia Jaber, Theresa Schwartz, Laura Crowley, Michael Crane, Shani Irby, Mayris P. Webber, Amit Verma, David J. Prezant

**Affiliations:** 1 Myeloma Service, Department of Medicine, Memorial Sloan Kettering Cancer Center, New York, New York; 2 Department of Medicine, Montefiore Medical Center, Bronx, New York; 3 Bureau of Health Services, Fire Department of the City of New York, Brooklyn, New York; 4 Department of Epidemiology and Population Health, Albert Einstein College of Medicine, Bronx, New York; 5 Division of Hemato-Oncology, Department of Oncology, Albert Einstein College of Medicine and Montefiore Medical Center, Bronx, New York; 6 Department of Laboratory Medicine, Memorial Sloan Kettering Cancer Center, New York, New York; 7 Department of Hematopathology, Memorial Sloan Kettering Cancer Center, New York, New York; 8 Division of Biostatistics, Department of Epidemiology and Population Health, Albert Einstein College of Medicine, Bronx, New York; 9 Mount Sinai School of Medicine, New York, New York; 10 Department of Medicine, Division of Pulmonary Medicine, Montefiore Medical Center and Albert Einstein College of Medicine, New York, New York

## Abstract

**Importance:**

The World Trade Center (WTC) attacks on September 11, 2001, created an unprecedented environmental exposure to known and suspected carcinogens suggested to increase the risk of multiple myeloma. Multiple myeloma is consistently preceded by the precursor states of monoclonal gammopathy of undetermined significance (MGUS) and light-chain MGUS, detectable in peripheral blood.

**Objective:**

To characterize WTC-exposed firefighters with a diagnosis of multiple myeloma and to conduct a screening study for MGUS and light-chain MGUS.

**Design, Setting, and Participants:**

Case series of multiple myeloma in firefighters diagnosed between September 11, 2001, and July 1, 2017, together with a seroprevalence study of MGUS in serum samples collected from Fire Department of the City of New York (FDNY) firefighters between December 2013 and October 2015. Participants included all WTC-exposed FDNY white, male firefighters with a confirmed physician diagnosis of multiple myeloma (n = 16) and WTC-exposed FDNY white male firefighters older than 50 years with available serum samples (n = 781).

**Exposures:**

WTC exposure defined as rescue and/or recovery work at the WTC site between September 11, 2001, and July 25, 2002.

**Main Outcomes and Measures:**

Multiple myeloma case information, and age-adjusted and age-specific prevalence rates for overall MGUS (ie, MGUS and light-chain MGUS), MGUS, and light-chain MGUS.

**Results:**

Sixteen WTC-exposed white male firefighters received a diagnosis of multiple myeloma after September 11, 2001; median age at diagnosis was 57 years (interquartile range, 50-68 years). Serum/urine monoclonal protein isotype/free light-chain data were available for 14 cases; 7 (50%) had light-chain multiple myeloma. In a subset of 7 patients, myeloma cells were assessed for CD20 expression; 5 (71%) were CD20 positive. In the screening study, we assayed peripheral blood from 781 WTC-exposed firefighters. The age-standardized prevalence rate of MGUS and light-chain MGUS combined was 7.63 per 100 persons (95% CI, 5.45-9.81), 1.8-fold higher than rates from the Olmsted County, Minnesota, white male reference population (relative rate, 1.76; 95% CI, 1.34-2.29). The age-standardized prevalence rate of light-chain MGUS was more than 3-fold higher than in the same reference population (relative rate, 3.13; 95% CI, 1.99-4.93).

**Conclusions and Relevance:**

Environmental exposure to the WTC disaster site is associated with myeloma precursor disease (MGUS and light-chain MGUS) and may be a risk factor for the development of multiple myeloma at an earlier age, particularly the light-chain subtype.

## Key Points

**Question:**

Are environmental exposures from the World Trade Center disaster site associated with multiple myeloma and its precursor disease, monoclonal gammopathy of undetermined significance (MGUS), in New York City firefighters?

**Findings:**

In this case series, 16 participants were diagnosed with multiple myeloma after September 11, 2001, with a median age of disease onset of 57 years, and in subsets with relevant data, a high proportion of the cases had light-chain myeloma, and plasma cells were CD20 positive. In the screening study, World Trade Center exposure was found to be statistically significantly associated with light-chain MGUS and overall MGUS.

**Meaning:**

World Trade Center disaster exposures are associated with myeloma precursor disease (MGUS) and may be a risk factor for the development of multiple myeloma at an earlier age.

## Teaser

This case series assesses whether environmental exposures from the World Trade Center disaster site are associated with multiple myeloma and its precursor disease, monoclonal gammopathy of undetermined significance, in New York City firefighters.

## Introduction

Multiple myeloma is a clonal neoplasm of differentiated B cells (plasma cells). It is one of the most common hematologic malignant neoplasms among adults, affecting approximately 100 000 persons currently living with the disease in the United States, and has an age-adjusted incidence rate of 6.53 per 100 000 per year.^1,2,3^ Multiple myeloma is most frequently diagnosed among people aged 65 to 74 years; only approximately 5% of cases are diagnosed before 50 years.^2^ In the general population, approximately 80% of patients with multiple myeloma have expression of IgH (referred to as “monoclonal-(M)-protein”) and 20% have abnormal light-chain proteins detectable in peripheral blood.^4^ Evidence from a large, prospective, population-based cancer screening trial shows that IgH-secreting and light-chain–secreting multiple myeloma are consistently preceded by their respective precursor states, monoclonal gammopathy of undetermined significance (MGUS) and light-chain MGUS, which can be detected in peripheral blood.^5^

Although the cause of multiple myeloma and its precursor conditions (MGUS and light-chain MGUS) remains largely unclear, previous studies have reported an increased risk among individuals exposed to known and suspected carcinogens including polychlorinated biphenyl (PCB), dioxins, polycyclic aromatic hydrocarbons (PAHs), and asbestos.^6,7,8^ The attacks on the World Trade Center (WTC) on September 11, 2001 (9/11), created an unprecedented environmental exposure to aerosolized dust and gases that contained known carcinogens including PCBs and PAHs.^9^ These substances were produced by the collapsed and burning buildings and by the diesel smoke emitted from heavy equipment used during the 10-month rescue and recovery effort. Recent cohort studies of first responders, construction workers, and volunteers at the WTC site provided evidence linking exposure to the WTC aerosolized dust and gases with multiple myeloma and other malignant neoplasms. In 2009, a case series (N = 8) suggested an excess of early-onset of multiple myeloma among WTC-exposed first responders in the General Responder Cohort; 4 of the individuals were 45 years or younger at diagnosis.^10^ Since 2011, studies have examined the post-9/11 incidence of multiple myeloma, and other cancers, in 3 WTC-exposed cohorts compared with the general population. A study of 55 778 New York state residents enrolled in the WTC Health Registry (including rescue workers, recovery workers, and those who lived or worked near the WTC) reported a nearly 3-fold (standardized incidence ratio [SIR], 2.85; 95% CI, 1.15-5.88) higher risk of multiple myeloma, based on 7 cases.^11^ However, a follow-up study focusing on 10-year cancer incidence patterns in the same population observed a nonsignificant 1.4-fold (SIR, 1.35; 95% CI, 0.70-2.36) increased risk of multiple myeloma.^12^ Similar studies among 8927 Fire Department of the City of New York (FDNY) WTC-exposed firefighters reported an SIR of 1.49 (95% CI, 0.56-3.97)^13^ while another among 20 984 non-FDNY responders from the General Responder Cohort reported an SIR of 1.41 (95% CI, 0.64-2.67).^14^

To improve our understanding of this association, we identified and characterized all WTC-exposed white, male firefighters from FDNY who received a diagnosis of multiple myeloma from September 12, 2001, to July 1, 2017. Second, we conducted a screening study for myeloma precursor disease among the FDNY subset of 781 white male WTC-exposed firefighters older than 50 years. The aim of our screening study was to define the age-specific prevalence of MGUS and light-chain MGUS in WTC-exposed New York City male firefighters and to compare the FDNY prevalence with that in the male Olmsted County, Minnesota, population. We also assessed patterns of myeloma precursor disease in relation to our exposure metric (time of initial arrival at the WTC site) to test for a possible exposure-response association.

## Methods

The case series and screening studies were approved by the Institutional Review Boards of Montefiore Medical Center and Albert Einstein College of Medicine. All participants provided written consent to research.

### Multiple Myeloma Case Series

#### Population and Case Information

**Figure 1.**
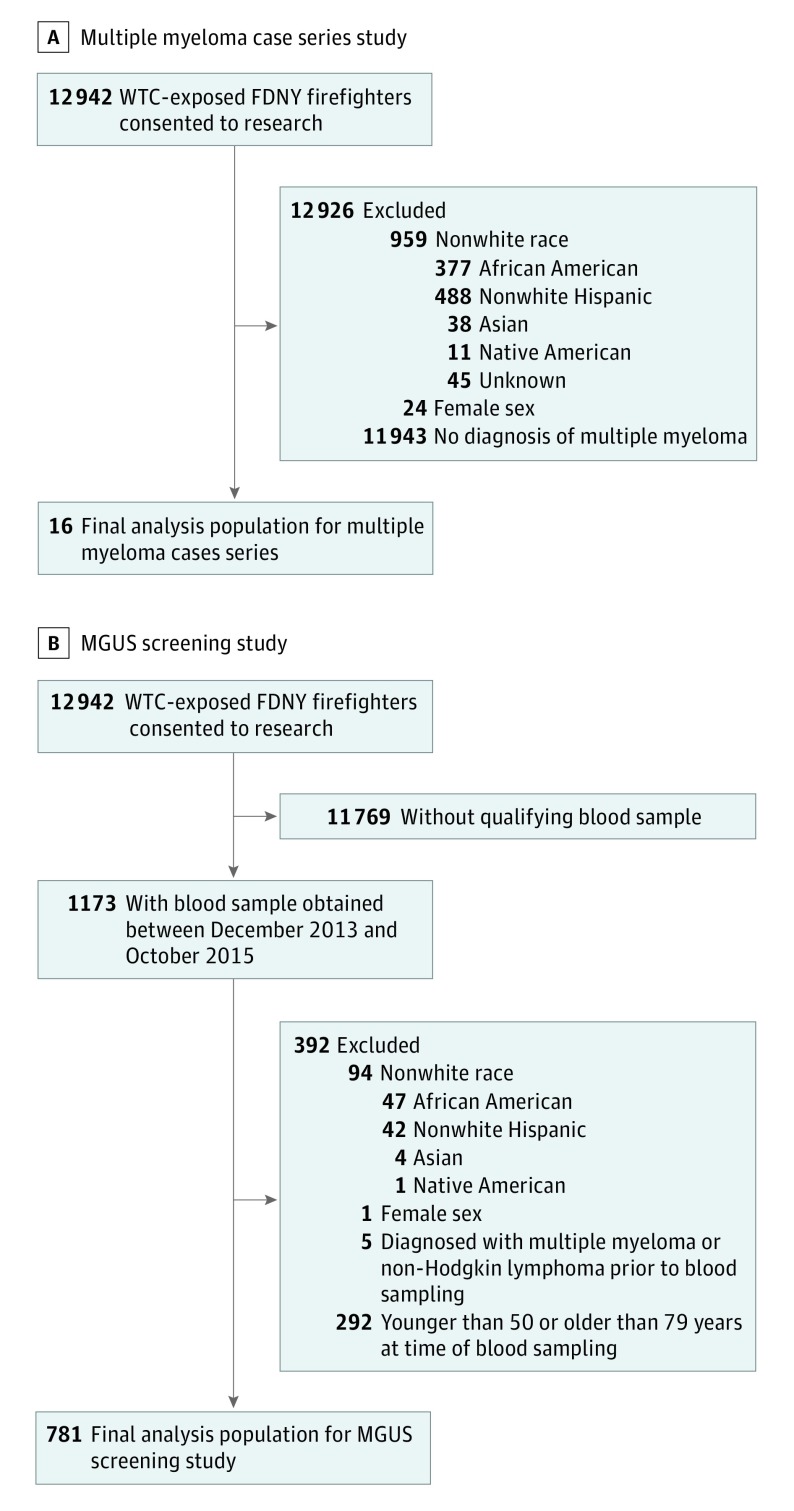
Exclusion Criteria FDNY indicates Fire Department of the City of New York; MGUS, monoclonal gammopathy of undetermined significance; WTC, World Trade Center.

Through the WTC Health Program, FDNY WTC-exposed firefighters (N = 12 942) receive comprehensive physical and mental health services. All white, male firefighters with a post-9/11 diagnosis of multiple myeloma (n = 16) in the FDNY WTC Health Program as of July 1, 2017, were included in the case series population (Figure 1A). The cases were confirmed in 2 ways: (1) via state tumor registry matches with New York, New Jersey, Connecticut, Pennsylvania, Florida, North Carolina, South Carolina, Arizona, and Virginia. A total of 8622 of 8830 (98%) retired FDNY firefighters in this cohort currently reside in these states. All active FDNY firefighters are required to live in New York City or neighboring New York state counties of Westchester, Rockland, Orange, Nassau, or Suffolk; (2) via FDNY WTC Health Program medical assessments/records reviewed by an experienced clinician (N.J.).^13^ Using medical records from the time of diagnosis, we extracted information on age at diagnosis, bone marrow aspirate and biopsy reports, and serum and urine protein testing. We performed complete case analysis when outcome data were missing.

### FDNY MGUS Screening Study

#### Population

The FDNY WTC Health Program provides regular monitoring examinations approximately every 12 to 18 months. From December 2013 through October 2015, serum samples were collected from 1173 WTC-exposed firefighters during routine monitoring examinations (Figure 1b). If a firefighter had more than 1 monitoring examination during the study period, serum was collected from the first examination. To facilitate comparison with our multiple myeloma results and our external comparison group, we restricted the analysis to white men. We excluded specimens from 5 who had received a diagnosis of myeloma or non-Hodgkin lymphoma prior to blood sampling. We also excluded participants younger than 50 or older than 79 years at the time of blood sampling. The final study cohort included 781 white, male, WTC-exposed firefighters (Table). All serum samples were analyzed for MGUS or light-chain MGUS in 2016.

**Table.** Characteristics of World Trade Center (WTC)-Exposed Fire Department of the City of New York (FDNY) White Male Firefighters in the Screening Study and Olmsted County, Minnesota, White Male Cohort

Characteristic	No. (%)	
	WTC-Exposed FDNY Firefighters (N = 781)	Olmsted County (N = 7612)
Age, y		
50-59	482 (61.7)	3450 (45.3)
60-69	225 (28.8)	2554 (33.6)
70-79	74 (9.5)	1608 (21.1)
WTC arrival date, 2001		
Morning of Sep 11	116 (14.9)	0
Afternoon of Sep 11	419 (53.6)	0
Sep 12	125 (16.0)	0
Sep 13-24	112 (14.3)	0
Later than Sep 24	9 (1.2)	0
Time spent at WTC site, mean (SD), mo	3.17 (2.68)	0

Demographic data including age at blood sample collection, race, and sex were obtained from the FDNY employee database. Additionally, data from the first self-administered health questionnaire, which began in October 2001, were used to categorize level of WTC exposure based on initial arrival time (arriving the morning of 9/11 [most highly exposed]; arriving the afternoon of 9/11; arriving September 12, 2001; arriving between September 13 and 24, 2001; and arriving between September 25, 2001, and July 24, 2002 [least exposed], when the WTC site closed) and duration at the WTC site (months in which a participant worked at least 1 day at the WTC site).

#### Serum Specimen and Laboratory Methods

We obtained a 0.5-mL aliquot for each study participant who consented to the FDNY research protocol. Each aliquot tube was labeled only with the participant’s coded identification number. All specimens were shipped on dry ice to the Protein Laboratory at Memorial Sloan Kettering Cancer Center, where protein assays were performed. The samples were tested concurrently and results were assessed by 2 of us (O.L. and K.M.) in a blinded fashion.^5,15,16,17^

#### Comparison Population: Olmsted County, Minnesota

We used published data from the population-based Olmsted County, Minnesota, study, the only available screening study including both MGUS and light-chain MGUS assays,^18^ as our comparison population. The racial distribution of the population in Olmsted County is predominantly white.^19^ We focused on the Olmsted County MGUS and light-chain MGUS rates in men 50 to 79 years old (N = 7612). Among the 7612 men, the prevalence of overall MGUS (ie, MGUS and light-chain MGUS), MGUS, and light-chain MGUS was 4.4% (n = 333), 3.4% (n = 258), and 1.0% (n = 75), respectively.^18^

### Statistical Analysis

The crude age-specific prevalence rates were calculated for white men as the total number of cases within each age stratum divided by the total number of individuals within that age stratum. Prevalence rates for overall MGUS, MGUS, and light-chain MGUS were calculated for the FDNY study population. Additionally, to enable external comparison, prevalence rates were age standardized to the US 2000 male population, ages 50 to 79 years. Age-adjusted 95% confidence intervals were calculated for directly standardized relative rates (RRs) using the modified γ approximation method, which assumes a Poisson distribution. Standard errors for 95% Mantel-Haenszel confidence limits of standardized relative risks were calculated using the Greenland and Robins^20^ variance formula. Participants older than 79 years were excluded from this analysis due to small numbers in the firefighting cohort. Exposure to the WTC, using time of arrival at the WTC site as a proxy for intensity, was evaluated separately in a stratified analysis. All analyses were performed using SAS, version 9.4, and R v.3.2.0.

## Results

### Multiple Myeloma Case Series

We identified 16 white male responders from the FDNY firefighter cohort with a post-9/11 diagnosis of multiple myeloma. The median age at diagnosis was 57 years (range, 38-76 years). The median time between 9/11 and diagnosis was 12.0 years (range, 1.0-15.7 years). The myeloma cell infiltration of the bone marrow ranged between less than 10% and 90% across individuals; immunophenotypic characterization of the bone marrow sample revealed CD20-positive plasma cells in 5 of 7 (71%; 95% CI, 36%-92%) tested cases. Results on serum and/or urine monoclonal proteins isotype and free light chains were available for 14 cases; 7 (50%; 95% CI, 27%-73%) had light-chain multiple myeloma. Individual-level data for the case series, including plasma-cell percentage, serum and/or urine monoclonal protein isotype and free light chains, and plasma-cell CD20 expression are provided in eTable 1 in the Supplement.

### MGUS Screening Study Demographic Characteristics

The Table provides selected demographic characteristics of the FDNY firefighter cohort and Olmsted County comparison population. The median age at time of FDNY specimen collection was 57 years (interquartile range, 54-62 years). The majority of firefighters arrived at the WTC site on the day of the attacks (535 [68.5%]) and spent a mean (SD) 3.17 (2.68) months working at the WTC site. The Olmsted County cohort was assumed to have no WTC exposure. The median age of firefighters with MGUS was nonsignificantly younger than that among the reference population: 62 years (FDNY firefighter cohort) vs 70 years (Olmsted County comparison population). Similarly, firefighters with light-chain MGUS were nonsignificantly younger than the reference population (median age, 61 vs 68 years). Specific characteristics of MGUS are found in eTable 2 in the Supplement.

### MGUS and Light-Chain MGUS Age-Standardized and Relative Rates

**Figure 2.**
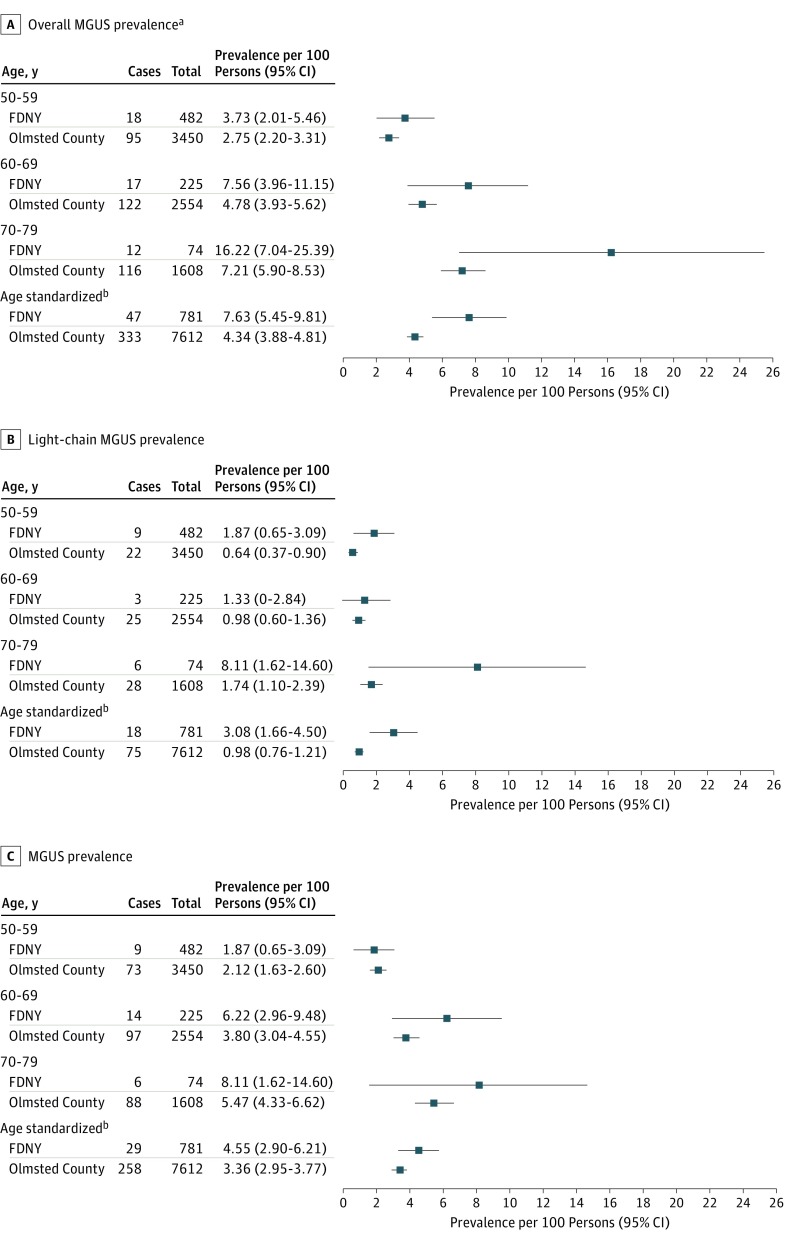
Prevalence of Monoclonal Gammopathy of Undetermined Significance (MGUS) and Light-Chain MGUS in World Trade Center–Exposed Fire Department of the City of New York (FDNY) White Male Firefighters and Comparison Population ^a^Overall MGUS includes both MGUS and light-chain MGUS cases. ^b^Prevalence rates are age standardized to US 2000 male population age 50 to 79 years.

Among white men aged 50 to 79 years, the age-standardized prevalence rate (ASR) of overall MGUS among WTC-exposed FDNY firefighters was 7.63 per 100 persons (95% CI, 5.45-9.81), which was 1.8-fold significantly higher compared with the rate among those from Olmsted County, Minnesota (ASR, 4.34 per 100 persons; 95% CI, 3.88-4.81 per 100 persons and RR, 1.76; 95% CI, 1.34-2.29) (Figure 2A). Figure 2B shows that, among the same population, the ASR of FDNY firefighters with only light-chain MGUS was 3.08 per 100 persons (95% CI, 1.66-4.50 per 100 persons), much greater than that of Olmsted County, which had an ASR of 0.98 per 100 persons (95% CI, 0.76-1.21 per 100 persons). The relative risk of having light-chain MGUS was 3.1-fold significantly higher (RR, 3.13; 95% CI, 1.99-4.93) when comparing WTC-exposed FDNY firefighters with the Olmsted County population. Last, Figure 2C shows that, among the same population, the ASR of FDNY firefighters with only MGUS was 4.55 per 100 persons (95% CI, 2.90-6.21 per 100 persons) and was slightly but not significantly higher than the Olmsted County cohort (ASR, 3.36; 95% CI, 2.95-3.77; RR, 1.35; 95% CI, 0.96-1.91) (Figure 2C). Overall MGUS was assessed by WTC arrival time and by duration at the WTC site; for all arrival times the ASRs were greater than in the reference population, although we did not find an exposure gradient (data not shown). Additionally, there was no significant difference in ASRs when duration of work at the WTC site was included in the analyses (data not shown).

## Discussion

In this first comprehensive study focusing on characteristics of multiple myeloma and its precursor disease (MGUS and light-chain MGUS) among WTC-exposed first responders, we observe striking patterns. We found the median age of multiple myeloma diagnosis to be 57 years, which is roughly 12 years younger than what is seen nationally,^2^ and because symptoms usually develop shortly after clinical manifestation of the disease (approximately 1 year), this would argue against a lead-time bias. Furthermore, the proportion of participants with CD20-expressing plasma cells—characteristics associated with a poorer prognosis—was more than 3.5-fold higher than found in other populations (71% vs approximately 20%).^2,5,21,22^ Among WTC-exposed white, male firefighters, the proportion of light-chain multiple myeloma was more than double that of the general population (50% vs approximately 20%).^4^ Similarly, in our screening study, we found a 2-fold significantly higher risk of myeloma precursor disease, particularly light-chain MGUS, the precursor of light-chain multiple myeloma.^5^ As suggested previously based on smaller numbers,^10,11,12,13,14^ our study shows that WTC exposure may be a risk factor for the development of multiple myeloma and its precursor disease.

Prior studies in cohorts without WTC exposure have found an increased risk of MGUS and light-chain MGUS among individuals exposed to known and suspected carcinogens including PCB, dioxins, PAHs, and asbestos,^6,7,8^ including exposure to Agent Orange (which contains the human carcinogen 2,3,7,8-tetrachlorodibenzo-p-dioxin).^6^ Interestingly, among veterans exposed to Agent Orange who were found to have myeloma precursor disease, there was a particular excess risk of light-chain MGUS (11 of 34 [32%] of the MGUS cases were light-chain MGUS).^6^

In our multiple myeloma case series, we found 7 of 14 (50%) cases to be of light-chain multiple myeloma subtype, which is more than double the rate in other populations (50% vs approximately 20%).^4^ In our screening study, we observed 18 of 47 (38%; 95% CI, 26%-53%) of the precursor cases to have light-chain MGUS, the precursor of light-chain multiple myeloma. Furthermore, we found that the risk of having light-chain MGUS was 3 times higher (RR, 3.13; 95% CI, 1.99-4.93) among our population compared with the Olmsted County population. These findings are of interest due to previously observed associations between light-chain multiple myeloma and light-chain MGUS and exposure to toxins^6,17^ and chronic immune stimulation.^23^

Many WTC first responders were initially exposed to aerosolized dust and toxic fumes from burning jet fuel and building materials. For the next 10 months of the rescue, recovery, and cleanup effort, responders were exposed to burning subterranean fires that released trapped gases and dust, and were not extinguished until the end of December. The final insult included diesel fuel combustion byproducts from heavy equipment used at the site. The WTC dust itself included pulverized cement, glass fibers, asbestos, lead, PAHs, PCBs, and polychlorinated furans and dioxins produced as combustion byproducts from the collapsed and burning buildings.^9^ Many of these substances are known carcinogens, providing biologic plausibility for an association between WTC exposure and cancer.^9,24,25,26^ Although some contaminants could directly cause cancer, WTC exposure has been shown to trigger chronic inflammation resulting in upper and lower respiratory diseases and autoimmune diseases,^27,28,29^ and, therefore, inflammation-induced oncogenesis should also be considered as a potential mechanism.^23^

Several surface antigens are used to characterize individual plasma cells as malignant or normal. For example, compared with normal plasma cells, abnormal plasma cells tend to be low in the expression of CD19 and CD27, have weaker expression of CD45, and increased expression of CD28, CD56, and CD117.^30^ Expression of CD20 is typically seen during the maturation process of B cells and absent from plasma cells; however, CD20 expression can be detected in 13% to 22% of patients with multiple myeloma diagnosed in the general population.^22^ Preliminary data suggest that subsets of CD20-positive multiple myeloma patients have a poorer prognosis.^5,21^ In the present study, albeit based on small numbers, immunophenotypic characterization revealed CD20-positive plasma cells in 5 of 7 (71%) multiple myeloma cases tested among WTC-exposed responders. Future work is needed to expand on these observations.

### Limitations

We acknowledge that our study has limitations. First, in our myeloma case series analysis, we were not able to rigorously compare FDNY and national age-adjusted incidence rates due to small or zero counts in certain age strata. This is often a challenge in the study of rare cancers.^31^ Additionally, when MGUS was assessed by WTC arrival time,^32^ for all arrival times the ASRs were greater than in the reference population. However, the study was underpowered to detect an exposure-response gradient association between WTC exposure and MGUS. Nonetheless, we did observe an elevated risk of overall MGUS and light-chain MGUS compared with the Olmsted County population. While a comparison group composed exclusively of firefighters with no exposure to the WTC disaster or a truly random sample of the US population would be most desirable, no such cohort data were available. Specifically, no other study meeting those criteria screened all participants for light-chain MGUS in the same manner that we did. The Olmsted County population matched our testing protocol and was demographically similar and thus provided a valuable comparison for this study. Similarly, due to small numbers in the firefighter cohort and lack of an adequate comparison population, races other than white and women were excluded. Future research investigating MGUS and light-chain MGUS may provide additional comparisons to our full population, as well as other WTC-exposed populations, important populations in need of follow-up.

Furthermore, in our case series analysis it is possible that the onset of precursor disease states may have preceded WTC exposure; however, 75% of myeloma cases were diagnosed more than 5 years after 9/11, with half being at least 12 years following the attacks. This suggests that, for most, the precursor disease likely developed after 9/11.^33^ Last, while we controlled for the main risk factors for MGUS, we cannot rule out the possibility of uncontrolled confounding between the FDNY population and the Olmsted County population.

This study had a number of strengths. First, we believe that our case ascertainment was excellent; this included matching to tumor registries where more than 98% of our cohort reside, as well as full access to the FDNY electronic medical record where approximately 87% have had a monitoring or treatment visit within the past 2 years. Second, to our knowledge, this is the largest study to characterize multiple myeloma in WTC-exposed responders. More importantly, this is the first study to establish the age-specific prevalence of MGUS/light-chain MGUS in a well-defined population of WTC-exposed responders.

## Conclusions

In summary, we identified and characterized all WTC-exposed white, male FDNY firefighters who received a diagnosis of multiple myeloma from September 12, 2001 to July 1, 2017, and 50% (7 of 14) of these cases were light-chain multiple myeloma. The median age at multiple myeloma diagnosis was 57 years, which is 12 years younger than what is observed in national data.^2^ A high proportion of patients with multiple myeloma had CD20-expressing plasma cells, which is a characteristic associated with a poorer prognosis.^5,21^ In the screening study including 781 white male WTC-exposed FDNY firefighters, we found the risk of overall MGUS to be 2-fold higher compared with the rate in the Olmsted County reference population; in particular, the risk of light-chain MGUS was higher, which may have important prognostic implications. Taken together, our results show that environmental exposure due to the WTC attacks is associated with myeloma precursor disease (MGUS and light-chain MGUS) and may be a risk factor for the development of multiple myeloma at an earlier age, particularly the light-chain subtype.

## Notes

Supplement. **eTable 1.** Post-9/11 multiple myeloma cases among WTC-exposed FDNY white male firefighters **eTable 2.** Myeloma precursor disease screening study: characteristics of MGUS and light-chain-MGUS in WTC-exposed FDNY white male firefighters
