# Supplementary material for: Multiple Myeloma and Its Precursor Disease Among Firefighters Exposed to the World Trade Center Disaster
Source: JAMA Oncol. 2018 Apr 26;4(6):821–7. doi: 10.1001/jamaoncol.2018.0509 (PMC6145680; doi:10.1001/jamaoncol.2018.0509)
Supplement: Supplement. — eTable 1. Post-9/11 multiple myeloma cases among WTC-exposed FDNY white male firefighters eTable 2. Myeloma precursor disease screening study: characteristics of MGUS and light-chain-MGUS in WTC-exposed FDNY white male firefighters [file jamaoncol-4-821-s001.pdf]

Supplementary Online Content

Landgren O, Rachel Zeig-Owens R, Giricz O, et al. Multiple myeloma and its precursor disease among firefighters exposed to the World Trade Center disaster. *JAMA Oncol*. Published online April 26, 2018. doi:10.1001/jamaoncol.2018.0509

eTable 1. Post-9/11 multiple myeloma cases among WTC-exposed FDNY white male firefighters

eTable 2. Myeloma precursor disease screening study: characteristics of MGUS and light-chain-MGUS in WTC-exposed FDNY white male firefighters

This supplementary material has been provided by the authors to give readers additional information about their work.

Open Access: This is an open access article distributed under the terms of the CC-BY License. © 2018 Landgren O, et al. *JAMA Oncology*.

eTable 1. Post-9/11 multiple myeloma cases among WTC-exposed FDNY white male firefighters

Patient number	Age at diagnosis	Plasma cell % in the bone marrow	Serum/urine monoclonal protein isotype and free light-chains	CD20 expression on plasma cells
1	58	40-50%	–*	Positive
2	59	10%	Free lambda light-chain	Positive
3	38	–*	Free lambda light-chain	ND
4	55	>15%	Free lambda light-chain	Negative
5	65	50%	Free kappa light-chain	ND
6	46	80-90%	IgG lambda & free lambda light-chain	Negative
7	56	20%	IgG kappa & free kappa light-chain	ND
8	74	80-90%	Free lambda light-chain	ND
9	49	10%	IgG kappa & free kappa light-chain	ND
10	46	–*	–*	ND
11	52	25%	IgG kappa	Positive
12	52	–*	Free kappa light-chain	ND
13	71	60-70%	IgG lambda & free lambda light-chain	Positive
14	60	80-90%	IgG lambda & free lambda light-chain	ND
15	69	>10%	IgG lambda & free lambda light-chain	Positive
16	76	<10%**	Free kappa light-chain	ND

Footnote: The WTC Health Program provide comprehensive physical and mental health services to WTC exposed. All responders from the FDNY databases with a post-9/11 diagnosis of multiple myeloma are included in the above case series population (until 7/1/2017). The information was obtained through review of medical records from the time of diagnosis.

Abbreviations: ND = not done; -* = data missing; **Extramedullary plasmacytoma, plasma cell percentage in the bone marrow <10%

Open Access: This is an open access article distributed under the terms of the CC-BY License. © 2018 Landgren O, et al. *JAMA Oncology*.

eTable 2. Myeloma precursor disease screening study: characteristics of MGUS and light-chain-MGUS in WTC-exposed FDNY white male firefighters

Characteristic of MGUS	WTC- exposed FDNY firefighters, (n=781)	
**MGUS**	n	%
Overall MGUS[a]	47	6.01
MGUS	29	3.71
IgG	17	2.18
IgA	6	0.77
IgM	5	0.64
Biclonal[b]	1	0.13
Light-chain-MGUS	18	2.30
**Concentration, Median (IQR)**		
M-protein g/dL[c]	0.37	0.21-0.59
Free-κ light chain, mg/dL[d]	2.32	2.12-2.74
Free-λ light chain, mg/dL[d]	1.23	1.09-1.38
dFLC, mg/dL[d]	1.15	0.95-1.65
**FLC-ratio, median (IQR)[d]**	1.86	1.70-2.19

Abbreviations:
dFLC: difference between involved and uninvolved free light chains
FLC: free light chain
IQR: Interquartile Range
MGUS: monoclonal gammopathy of undetermined significance

[a-] Includes MGUS and light-chain-MGUS cases
[b-] One firefighter had 2 separate bands of IgG
[c-] Based on 29 firefighters whose M-protein concentration was quantifiable
[d-] Based on 18 firefighters who had light-chain-MGUS as described in the methods section

Open Access: This is an open access article distributed under the terms of the CC-BY License. © 2018 Landgren O, et al. *JAMA Oncology*.

Footnote: To rule out MGUS and light-chain-MGUS, all serum specimens were first analyzed using conventional agarose-gel electrophoresis to determine the occurrence and pattern of M-protein bands, as described previously.[17] Samples observed with an M-protein band, equivocal band pattern, or abnormal free light-chain-(FLC)-ratio, were further analyzed by immunofixation to characterize the heavy- and light-chain isotypes of the M protein. Serum protein electrophoresis and immunofixation were performed using the SPIFE 3000 (Helena Laboratories). The FLC levels in all serum specimens were determined using a turbidimetric assay (Freelite; The Binding Site) performed on a SPAPLUS Automated Analyzer for Specialist Protein Analysis.[16] The Freelite assay (also used for the Olmsted County study that served as our external control) comprises 2 separate measurements: one to detect free-κ light chains (normal range, 3.3-19.4 mg/L), and the other to detect free-λ light chains (normal range, 5.7-26.3 mg/L).[15] We assessed monoclonality based on the κ/λ FLC-ratios.[16] MGUS cases were defined as having M-protein bands on SPEP and an M-protein concentration less than 30g/L.[5] Light-chain-MGUS was defined as the presence of an FLC band without heavy-chain expression in immunofixation or presence of an abnormal FLC-ratio (normal reference: 0.26-1.65) with an increased level (above the upper limit of normal) of the involved light chain.[5,15]

Open Access: This is an open access article distributed under the terms of the CC-BY License. © 2018 Landgren O, et al. *JAMA Oncology*.
